# The effect of a helmet type, home-use low-level light therapy device for chemotherapy-induced alopecia: study protocol for a randomized controlled trial

**DOI:** 10.1186/s13063-023-07823-x

**Published:** 2023-12-05

**Authors:** Cong-Xian Wu, Cheng-Hsin Li, Yi-Hsien Shiao, Huan-Yu Cheng, Tsung-Han Wu, Chun-Hui Lee, Zi-Yu Chang, Yuan-Chieh Yeh

**Affiliations:** 1 Department of Traditional Chinese Medicine, Chang Gung Memorial Hospital, Keelung Medical Center, Keelung, 204201 Taiwan; 2 Graduate Institute of Natural Products, Chang Gung University, Taoyuan, 333323 Taiwan; 3 The Institute of Health Policy and Management, National Taiwan University, Taipei, 106319 Taiwan; 4 Division of Hemato-oncology, Department of Internal Medicine, Chang Gung Memorial Hospital, Keelung, 20401 Taiwan; 5 College of Medicine, Chang Gung University, Taoyuan, 33320 Taiwan; 6 Division of General Surgery, Department of Surgery, Chang Gung Memorial Hospital, Keelung Medical Center, Keelung, 204201 Taiwan; 7 Institute of Traditional Medicine, School of Medicine, National Yang Ming Chiao Tung University, Taipei, 112304 Taiwan; 8 Program in Molecular Medicine, College of Life Sciences, National Yang Ming Chiao Tung University, Taipei, 112304 Taiwan

**Keywords:** Low-level light therapy, Chemotherapy-induced alopecia, Phototrichogram, Quality of life, Self-esteem, Chemotherapy-induced alopecia distress

## Abstract

**Background:**

Alopecia is one of the most common adverse effects of chemotherapy. It reduces the patient’s self-esteem and quality of life and the effect of therapy. Scalp cooling is the only verified current method for prevention but success is not guaranteed, particularly after receiving anthracycline-based combinations.

Low-level light therapy has been clinically proven to inhibit the progress of androgenic alopecia. A previous study using human subjects shows limited benefits for low-level light therapy for patients who suffer chemotherapy-induced alopecia but an increase in the number of probes and the optimization of light sources may improve the efficacy. This study determines the efficacy of low-level light therapy for the prevention of chemotherapy-induced hair loss for patients with breast cancer using a randomized controlled trial.

**Methods:**

One hundred six eligible breast cancer patients were randomly distributed into a low-level light therapy group and a control group, after receiving chemotherapy. Subjects in the low-level light therapy group received 12 courses of intervention within 4 weeks. Subjects in the control group received no intervention but were closely monitored.

The primary outcome is measured as the difference in the hair count in a target area between the baseline and at the end of week 4, as measured using a phototrichogram (Sentra scalp analyzer). The secondary outcomes include the change in hair count at the end of week 1, week 2, and week 3 and hair width at the end of week 1, week 2, week 3, and week 4, as measured using a phototrichogram, and the change in distress, the quality of life, and self-esteem due to chemotherapy-induced alopecia, at the end of week 4, as measured using a questionnaire.

**Discussion:**

This study improves cancer patients’ quality of life and provides clinical evidence.

**Trial registration:**

Registered at ClinicalTrials.gov—NCT05397457 on 1 June 2022.

**Supplementary Information:**

The online version contains supplementary material available at 10.1186/s13063-023-07823-x.

## Background

Chemotherapy-induced alopecia (CIA) is one of the most common side effects of chemotherapy. The incidence of CIA is 65% during an intervention [1]. Hair loss is often resolved within 3 to 6 months [2], but some patients experience prolonged or permanent alopecia after receiving standard-dose chemotherapy [3–7]. CIA also adversely affects patients in terms of social relationships, sexuality [1, 8], self-esteem, and quality of life [9]. Female patients with alopecia experience particular problems with self-esteem and can become sociophobic because CIA marks them as cancer patients, which affects their lives. Sixty-three percent of patients with CIA also admit to having problems with their career [10–12]. Therefore, CIA is a cosmetic and a socioeconomic issue.

Chemotherapy agents mostly damage the anagen matrix, which can create a dystrophic anagen pathway [13]. To reduce cellular drug uptake, scalp cooling is used to prevent alopecia but success is not guaranteed, especially after receiving anthracycline-based regimens [14, 15].

Low-level light therapy (LLLT) is a therapeutic method for treating hair loss that is non-invasive, brief, and easy to use. The mechanism for prevention of alopecia increases the anagen phase of hair and prevents hair from prematurely entering the catagen phase [16–18]. At the molecular level, cytochrome C oxidases, which are chromophores in the mitochondria of the skin cells, significantly affect this process. Cytochrome C oxidases absorb red and near-infrared light and light-induced nitric oxides are released from cytochrome C oxidases, which increase enzyme activity, electron transport, and the production of adenosine triphosphate and reactive oxygen species [19, 20]. Cytokines, such as vascular endothelial growth factor (VEGF) and inflammatory mediators, are then produced in greater amounts so vascularization is increased and hair follicle stem cell growth is stimulated [20].

A randomized, double-blind, sham device-controlled multicenter trial in 2013 by Hyojin Kim et al. determined the efficacy of LLLT for the treatment of androgenetic alopecia. After 24 weeks, improvements in hair density and hair diameter within the LLLT group are greater than those for the sham device group, and the results are statistically significant, with no serious adverse reactions [21].

In terms of CIA, in vitro evidence shows that LLLT affects mitochondria by up-regulating the anti-apoptotic proteins and preventing stem cells from entering apoptosis after receiving chemotherapy [22]. However, few studies use LLLT to treat CIA, and the results do not constitute significant clinical evidence. Only one study determines the effect of LLLT on CIA using human subjects, and the results show that LLLT gives patients with CIA improved hair counts, an increase in the amount of hair increase, and longer hair length [23]. However, this study uses fewer probes (laser diodes, LDs; light-emitting diodes, LEDs) in the helmet-type LLLT than the helmet that was used by Hyojin Kim [21].

To determine the effect of LLLT for treating CIA, this study conducts a clinical trial using a more advanced device with 69 LDs. The light source for LD is coherent but a LED is an incoherent light source, so LDs deliver energy to tissue more efficiently than LEDs [24, 25]. This randomized controlled trial determines whether the latest equipment produces better consequences for the treatment of CIA to ascertain whether it increases a patient’s chances of recovery.

## Methods

### Study design

This protocol conforms with the SPIRIT 2013 (Standard Protocol Items: Recommendations for Interventional Trials) statement (see Additional file 1 for the SPIRIT Checklist). There is no public or patient involvement in the design of the protocol. This study is a two-arm, randomized, controlled trial to determine the efficacy of LLLT for the treatment of CIA. Patients with breast cancer at Keelung Chang Gung Memorial Hospital, Taiwan, were screened by a general surgeon or an oncologist to diagnose and determine the severity of chemotherapy-induced alopecia. The diagnosis used the Common Terminology Criteria for Adverse Events [26].

After confirmation of alopecia, patients were referred to the department of Chinese medicine. The eligibility criteria were verified during the prior assessment, which was standardized by the outpatient department. If the patient was eligible and was willing to participate in the study, their full name and phone number was given to one of the study assessors to schedule a baseline assessment. One hundred six eligible participants were given a baseline assessment. At the time of the baseline visit, a consent form was completed by the participants or a legal guardian, as required by the Chang Gung Medical Foundation Institutional Review Board. A total of 106 participants were randomly distributed into two groups, with 53 individuals in each group:

- LLLT group: 12 courses of low-level light therapy over 4 weeks at Keelung Chang Gung Memorial Hospital, Taiwan
- Control group: no intervention but close monitoring (12 interviews within 4 weeks) at Keelung Chang Gung Memorial Hospital, Taiwan

**Fig. 1:**
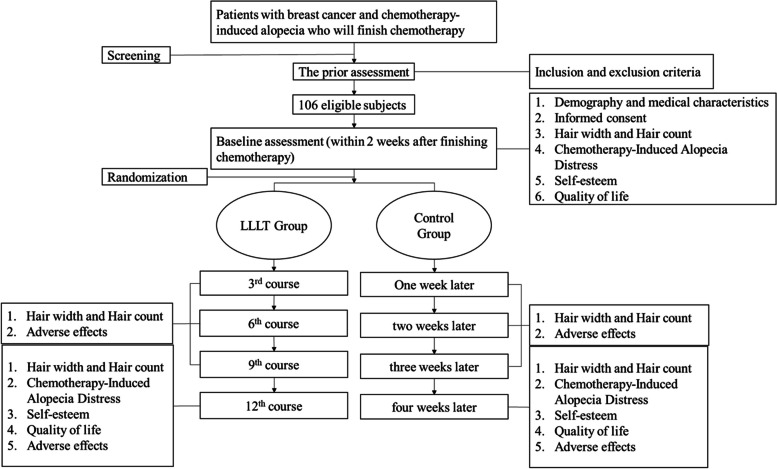
Flowchart. Boxes display the number of participants recruited for the clinical trial and those assigned to each group

The primary outcome is the difference in the hair counts for a target area between the baseline and the end of week 4. The secondary outcomes include the change in hair count at the end of week 1, week 2, and week 3 and hair width at the end of week 1, week 2, week 3, and week 4 and the change in distress, quality of life, and self-esteem due to chemotherapy-induced alopecia at the end of week 4. The flowchart for the study is shown in Fig. 1 and Table 1.

**Table 1:** Schedule of enrollment, intervention, and assessment

	Prior assessment	Baseline assessment	1 week later	2 weeks later	3 weeks later	4 weeks later
Patients						
➢ Inclusion and exclusion criteria	X	X				
➢ Informed consent		X				
➢ Demographics		X				
➢ Medical characteristics		X				
➢ Randomization and allocation concealment		X				
Primary outcome						
➢ Hair counts		X				X
Secondary outcomes						
➢ Hair width		X	X	X	X	X
➢ Hair counts			X	X	X	
➢ Chemotherapy-induced alopecia distress		X				X
➢ Self-esteem		X				X
➢ Quality of life		X				X
Adverse events			X	X	X	X

The table reveals the events the participants will encounter in the study. Each row represents each item. Each column represents the timeline. The letter X in this table represents that the participants will undergo the item at a specified time

Ten attending physicians in the Chinese Medicine Department at Keelung Chang Gung Memorial Hospital were involved in patient recruitment. Ten trained nurses assisted the attending physicians in providing patients with information sheets during the initial assessment. A trained research nurse supervised completion of the consent form during the baseline assessment. Five resident physicians conducted baseline and follow-up assessments, using a phototrichogram, determining any adverse effects and helping patients complete the questionnaires. An external statistician randomized the groups and ensured that the allocation was confidential. The eight individuals who were initially involved in the protocol design attended meetings every 3 months to discuss the progress of the study. An independent data monitoring expert and Institutional Review Board members conducted an annual audit. A coordinator was assigned to ensure the integrity of each dataset and two interns participated in collecting data for the study.

### Approval and registration

The procedures and consent form were approved by the Chang Gung Medical Foundation Institutional Review Board (protocol no. 202200395A3), and the audit was conducted once a year by an independent data monitoring expert and Chang Gung Medical Foundation Institutional Review Board. This study is also registered at ClinicalTrials.gov (NCT05397457) (Table 2).

**Table 2:** Trial registration data

Data category	Information
Primary registry and trial identifying number	ClinicalTrials.gov NCT05397457
Date of registration in primary registry	19 May, 2022
Secondary identifying numbers	202200395A3
Source(s) of monetary or material support	Nil
Primary sponsor	Chang Gung Memorial Hospital
Secondary sponsor(s)	Nil
Contact for public queries	Y.C.Y., MD [b9005030@gmail.com]
Contact for scientific queries	Y.C.Y., MD Department of Traditional Chinese Medicine, Chang Gung Memorial Hospital, Keelung Medical Center
Public title	Effect of Helmet Type, Home-use Low-level Light Therapy Device for Chemotherapy-induced Alopecia
Scientific title	*Effect of helmet type, home-use low-level light therapy device for chemotherapy-induced alopecia: study protocol for a randomized controlled trial*
Countries of recruitment	Taiwan
Health condition(s) or problem(s) studied	Breast cancer, chemotherapy-induced alopecia
Intervention(s)	Active comparator: low-level light therapy
	Control: no intervention
Key inclusion and exclusion criteria	Ages eligible for study: 20~75 years Sexes eligible for study: female Accepts healthy volunteers: no
	Inclusion criteria: female patients aged between 20-75 years with breast cancer, completing chemotherapy no more than 2 weeks, receiving chemotherapeutic agents containing taxanes, anthracyclines, or fluoropyrimidine, chemotherapy-induced alopecia classified into grade 2 in Common Terminology Criteria for Adverse Events, a life expectancy of at least 6 months
	Exclusion criteria: any serious mental illness or history, taking psychotropic drugs, taking any of the following medications for 6 months before initiation of the study: topical minoxidil, spironolactone, or topical calcitriol, receiving scalp cooling during chemotherapy, a medical history of dermatosis, scalp tumor, or melanoma, severe liver or kidney damage, pregnancy or potential pregnancy
Study type	Interventional
	Allocation: randomized intervention model Parallel assignment masking: none
	Primary purpose: treatment
	Phase: not applicable
Date of first enrollment	August 17, 2022 [Actual]
Target sample size	106
Recruitment status	Recruiting
Primary outcomes	Hair counts recorded by Sentra scalp analyzer [time frame: baseline; not designated as safety issue] Hair counts recorded by Sentra scalp analyzer [time frame: 12th course of intervention (four weeks after the first course of intervention); not designated as safety issue]
Key secondary outcomes	Hair counts recorded by Sentra scalp analyzer [time frame: 3rd course of intervention (four weeks after the first course of intervention); not designated as safety issue] Hair counts recorded by Sentra scalp analyzer [time frame: 6th course of intervention (four weeks after the first course of intervention); not designated as safety issue] Hair counts recorded by Sentra scalp analyzer [time frame: 9th course of intervention (four weeks after the first course of intervention); not designated as safety issue] Hair width recorded by Sentra scalp analyzer [baseline; not designated as safety issue] Hair width recorded by Sentra scalp analyzer [time frame: 3rd course of intervention (four weeks after the first course of intervention); not designated as safety issue] Hair width recorded by Sentra scalp analyzer [time frame: 6th course of intervention (four weeks after the first course of intervention); not designated as safety issue] Hair width recorded by Sentra scalp analyzer [time frame: 9th course of intervention (four weeks after the first course of intervention); not designated as safety issue] Hair width recorded by Sentra scalp analyzer [time frame: 12th course of intervention (four weeks after the first course of intervention); not designated as safety issue] Chemotherapy-Induced Alopecia Distress Scale [baseline; not designated as safety issue] Chemotherapy-Induced Alopecia Distress Scale [time frame: 12th course of intervention (four weeks after the first course of intervention); not designated as safety issue] Rosenberg Self-esteem Scale [baseline; not designated as safety issue] Rosenberg Self-esteem Scale [time frame: 12th course of intervention (four weeks after the first course of intervention); not designated as safety issue] European Quality of Life 5 Dimensions 5 Level Version [baseline; not designated as safety issue] European Quality of Life 5 Dimensions 5 Level Version [time frame: 12th course of intervention (four weeks after the first course of intervention); not designated as safety issue] European Organization for the Research and Treatment of Cancer Quality of Life Questionnaire [baseline; not designated as safety issue] European Organization for the Research and Treatment of Cancer Quality of Life Questionnaire [time frame: 12th course of intervention (four weeks after the first course of intervention); not designated as safety issue]

### Participants

One hundred six eligible patients were recruited at Keelung Chang Gung Memorial Hospital, Taiwan. Inclusion criteria are as follows: (1) female patients aged between 20 and 75 years with breast cancer; (2) completing chemotherapy no more than 2 weeks previously; (3) receiving chemotherapeutic agents containing taxanes, anthracyclines, or fluoropyrimidine; (4) presence of chemotherapy-induced alopecia that is classified as grade 2 in the Common Terminology Criteria for Adverse Events [26]; and (5) a life expectancy of at least 6 months.

Exclusion criteria include the following: (1) any serious mental illness or history; (2) taking psychotropic drugs; (3) taking any of the following medications for 6 months before initiation of the study: topical minoxidil, spironolactone, or topical calcitriol; (4) receiving scalp cooling during chemotherapy; (5) a medical history of dermatosis, scalp tumor, or melanoma; (6) severe liver or kidney damage; and (7) pregnancy or potential pregnancy.

Eligible patients participated in this study after completing the consent form, which asked if patients agreed to the use of their data, should they choose to withdraw from the trial. Participants’ permission was sought to share the relevant data with project-related individuals, including experts from universities or regulatory authorities. This trial does not involve collecting biological specimens for storage. All participants were closely monitored during the study. There is no anticipated harm for participants in this clinical trial, and no compensation was provided. Post-trial care is described in the following.

- In the event of any adverse reactions or damage due to the trial, the Chang Gung Memorial Hospital and the principal investigator, Yuan-Chieh Yeh, who is deputy director, assistant professor, provided compensation. There is no compensation for expected adverse reactions, as stated in the consent form.
- If any adverse reactions or damages, including adverse effects mentioned in the consent form, occurred due to the trial, the Chang Gung Memorial Hospital provided professional medical care and consultation at no cost to the participants.
- Except for the above, no other forms of compensation were provided. If participants were not willing to accept these risks, they were advised not to participate in the trial.
- Signing the consent form did not result in any loss of legal rights for participants.

### Sample size calculation

A realistic difference was used to calculate the sample size. The calculation uses an 80% statistical power and a significance level (alpha error) of 5%. Similar to the study by Stadler in 2021 [23], the calculated effect size (Cohen’s *d*) was approximately 0.54, which corresponds to a medium effect. This calculation gives a total sample size of 88 individuals, but for an expected dropout rate of 20%, the revised requirement is 106 participants. All calculations used the G*Power software version 3.1.9.4 [27] to ensure the accuracy and consistency of the methodology. To increase our pool of participants, referrals were taken from general surgeons and oncologists and posters were used for recruitment.

### Randomization and allocation concealment

The statistician, who was not involved in participant recruitment, assessment, or treatment, devised the randomization schedule using a computer-generated random number table. Equal numbers of participants were allocated to each one of the two groups to give 53 in each group. The randomization code was only released after a patient had been recruited for the trial, when all the necessary baseline measurements had been completed.

Each envelope in the randomization list contains a randomization code for either the therapy group or the control group. The attending physician opened an envelope to determine the treatment condition to be administered, based on the information provided. The randomization code was hidden from assessors, the coordinator, data collectors, and statisticians, but because the manufacturer cannot provide a sham device that emits a red light without any LD, patients in the control group were not blinded. Assessors, the coordinator, data collectors, and the external statistician were also not blinded, but the assessor performed the assessment objectively. After the baseline assessment, the Chinese medical physician informed the participant of the type, time, and frequency of treatment. The external statistician analyzed data.

### Low-level light therapy

A helmet-type LLLT device was used by Hyojin Kim et al. and developed by Won Technology (Daejeon, Korea) [21]. The light sources for the device for this study are laser diodes (LDs) that emit 650 nm (5 mW, 69 units). All diodes run simultaneously through six cycles. Each cycle is composed of 2 min 50 s on and 10 s off so the energy per unit for each light source with Orion-PD ROHS (Ophir Optronics Ltd., Jerusalem, Israel) is 5 mW (± 20%) for LD. The total energy density for the device is 59 mW/cm^2^. Energy fluence is 60.43 J/cm^2^ for 18 min of treatment and 70.50 J/cm^2^ for 21 min of treatment. Participants in the LLLT group used the device three times per week for 4 weeks.

### Assessment procedures

#### Efficacy assessment

The primary outcome is the difference in the hair count for the target area between the baseline and the end of week 4, which was measured using a phototrichogram (Sentra scalp analyzer, R.O.C. Pat.N523148). The secondary outcomes include a change in in hair count at the end of week 1, week 2, and week 3 and hair width at the end of week 1, week 2, week 3, and week 4, as measured using a phototrichogram, and the change in distress due to chemotherapy-induced alopecia, quality of life, and self-esteem at the end of week 4, as measured using a questionnaire. The data that was collected by this study is administered only by the authors of the study.

Before the study, the assessors were trained to use a phototrichogram and in the use of questionnaires. The training consisted of reading the manual for the phototrichogram and questionnaires, discussion of any doubts, and a trial for one patient, which was performed by each assessor.

#### Phototrichogram Assessment

**Fig. 2:**
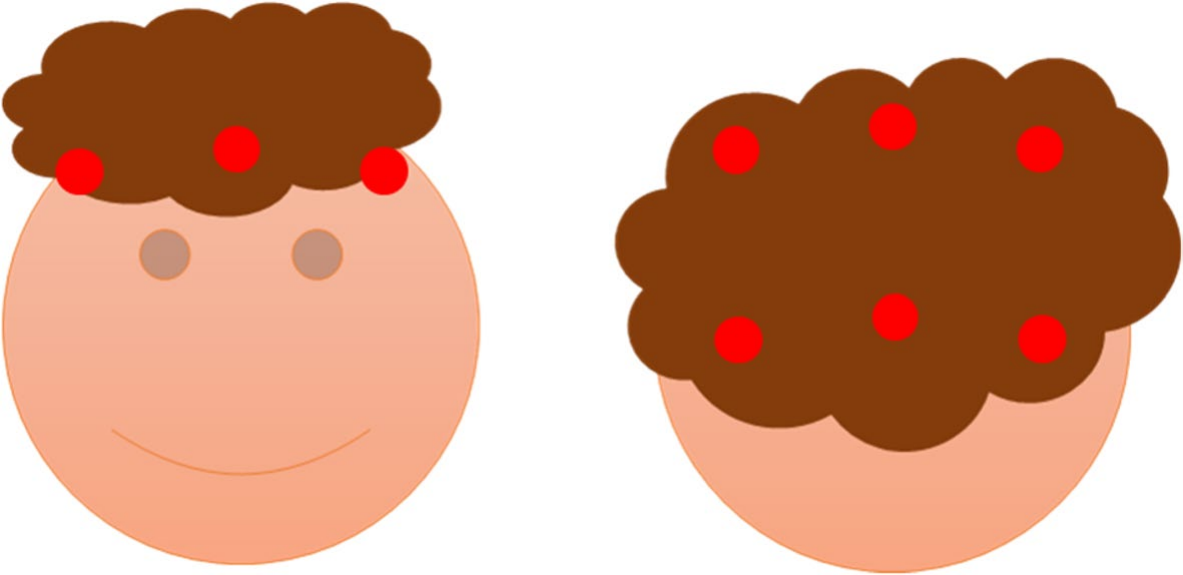
Nine areas where we measure hair counts and hair width. The points in this figure are the places where we measure hair counts and hair width

A Sentra scalp analyzer is an innovative phototrichogram that uses trichoscopy, image capture, and image interpretation to measure hair counts and hair width in 9 areas of 14.47 mm^2^, including the frontal, parietal, and occipital regions (Fig. 2). The target area is the place where hair loss is most prominent. Fifteen minutes were required to complete each assessment.

#### Distress due to chemotherapy-induced alopecia

A chemotherapy-induced alopecia distress scale is used [28]. The respective minimum and maximum scores for the chemotherapy-induced alopecia distress scale are 17 and 68, including 17 items with a 4-point Likert scale for 4 factors (physical, emotional, activity, and relationship). A higher value signifies greater chemotherapy-induced distress for the participant. The questionnaire was translated and submitted for cross-cultural adaption to Chinese [29]. Ten minutes were allotted to finish the questionnaire.

#### Quality of life

Quality of life is assessed using the validated Chinese version of the European Quality of Life 5 Dimensions 5 Level Version (EQ-5D-5L) [30, 31] and the European Organization for the Research and Treatment of Cancer Quality of Life Questionnaire (EORTC-QLQ-C30) [32–35].

The EQ-5D-5L is a generic health-related quality of life (HRQoL) instrument with five dimensions, including mobility, self-care, usual activities, pain/discomfort, and anxiety/depression. Each dimension has 5 levels: no problems, slight problems, moderate problems, severe problems, and extreme problems.

EORTC-QLQ-C30 is an integrated system for assessing the HRQoL of cancer patients participating in international clinical trials. The 30-item questionnaire is composed of both multi-item scales and single-item measures. These include five functional scales, three symptom scales, a global health status/QoL scale, and six single items. All scales and single-item measures have score of 0 to 100. A high score for a functional scale represents a high/healthy level of functioning. A high score for the global health status/QoL represents a high QoL. A high score for a symptom scale/item represents a high level of symptomatology/problems. All participants were given 15 min to complete these questionnaires.

#### Self-esteem

Self-esteem is measured using the Chinese version of the Rosenberg Self-esteem scale with 10 items [36]. Each item is scored on a 4-point Likert self-report scale, from 0 to 3. The total score is the sum of all item scores. A high score indicates high self-esteem. All participants were given 5 min to complete the questionnaire.

### Statistical analysis

Prior to statistical analysis, the data was imported into a spreadsheet and checked randomly by one of the authors, who was not involved in entering the data into the spreadsheet, to confirm whether there was any error in the transcription of the data. Statistical analysis uses SPSS v.15 for Windows. The baseline demographic and medical characteristics of participants for the study is analyzed using appropriate descriptive statistics for the two groups. To account for potential baseline confounding variables, analyses are adjusted. These variables include demographic and medical characteristics, particularly breast cancer staging and the dose and regimen for chemotherapy.

Univariate analysis is used to determine the association between the two variables, without adjusting for any confounding variables. Multivariable analysis is used to determine the relationship between the exposure and outcome variables while controlling for the effects of the confounding variables. A sensitivity analysis determines the robustness of the results to different modeling assumptions and to the inclusion or exclusion of certain variables. Intention-to-treat (ITT) analysis is used.

The primary outcome, which is the difference in hair count between the baseline and the end of week 4, is analyzed using an independent-sample *t*-test and the mean, standard deviation (SD), and 95% confidence interval are reported. Generalized estimating equations (GEEs) are used to determine any improvement in hair count and hair width at the end of week 1, week 2, week 3, and week 4. The change in CIA distress, self-esteem, and QoL at the end of week 4 is determined using an independent-sample *t*-test. Adverse events are analyzed using a two-sided Fisher’s exact test.

### Subgroup analysis

The homogeneity of the low-level light therapy effects on the primary outcome is determined using a one-way ANOVA (analysis of variance) with predefined subgroups, including whether the patient has been in menopause, immunohistochemical diagnosis (ER, PR, HER-2), and particularly the dose and regimen for hormone therapy. The study determines whether hormone therapy is related to recovery from chemotherapy-induced alopecia.

### Monitoring

There are no reports on the adverse effects of LLLT. The assessors monitored each case to decide whether treatment should be terminated. The project management group also held meetings every three months to review the progress of participants, determine the cause of any withdrawals, determine whether the study flowchart is to be revised, and determine if additional participants were needed to account for withdrawals. In the event of a withdrawal, the assessor contacted the participant by phone to inquire whether any adverse effects were experienced. The assessor also explained the benefits of LLLT and encouraged the participant to continue if the side effects were tolerable. An audit was conducted once a year by an independent data monitoring expert and the Chang Gung Medical Foundation Institutional Review Board. During the audit, in order to objectively assess the risk of the intervention, members of the Chang Gung Medical Foundation Institutional Review Board randomly contacted the participants to confirm whether the participants were well informed by the investigators and whether there were any adverse effects. If there was any doubt, the project was terminated.

### Trial status

Ethics approval was obtained in May 2022 from Chang Gung Medical Foundation Institutional Review Board. The trial was registered on 1 June 2022 (registration number: NCT05397457). Recruitment and training of the assessor began in May 2022. Recruitment of participants began on August 17, 2022. The project will be completed in August of 2025. The statistical analysis will be completed by the end of October 2025.

## Discussion

CIA affects a patient physiologically and emotionally because quality of life is reduced. Scalp cooling is the only management that is approved by the U.S. Food and Drug Administration for prevention. However, success is not guaranteed, particularly for patients receiving anthracycline-based combinations [14, 15]. There are also concerns about the therapy [37, 38].

The severity of hair follicle stem cell damage depends on the reversibility of hair loss [13]. An in vitro study showed that LLLT prevents 5-FU-treated mesenchymal stem cells from entering apoptosis due to the up-regulation of anti-apoptotic protein [22].

One study shows that LLLT does not improve hair count, hair increase, or hair length [23]. However, the number of probes and the type of light source has a significant effect on the efficacy of the device. Unlike LEDs, LDs are coherent light sources, which efficiently transfer energy to tissues [24, 25]. A device for which the light sources are all LDs is used by this study to improve outcomes.

One limitation of this study is that it does not use a sham device to eliminate the placebo effect. The manufacturer cannot provide a sham device that emits a red light without any LD, so it is impossible to blind the patients in the control group. The patients know the group to which they belong. To increase objectivity, a phototrichogram is used to measure the change in hair count and hair width. Another limitation is the short follow-up duration. It takes at least 3–6 months to recover from hair loss due to chemotherapy, but this study only records results for 4–6 weeks, so the results for the control group are temporary and may improve over time.

### Supplementary Information

**Additional file 1.**

## Data Availability

Due to ethical considerations, the datasets that are generated in the course of this study are not publicly available. However, interested parties may request access to the full study protocol, statistical code, and participant-level datasets from the corresponding author, subject to reasonable requirements.

## Abbreviations

CIA: Chemotherapy-induced alopecia
LLLT: Low-level light therapy
VEGF: Vascular endothelial growth factor
LDs: Laser diodes
LEDs: Light-emitting diodes
EQ-5D-5L: European Quality of Life 5 Dimensions 5 Level Version
EORTC-QLQ-C30: European Organization for the Research and Treatment of Cancer Quality of Life Questionnaire
HRQoL: Health-related quality of life
QoL: Quality of life
ANOVA: Analysis of variance
ITT: Intention-to-treat
GEEs: Generalized estimating equations
